# Optical behavior and marginal discoloration of a single shade resin composite with a chameleon effect: a randomized controlled clinical trial

**DOI:** 10.1038/s41405-024-00184-w

**Published:** 2024-02-20

**Authors:** Ruba Salah Anwar, Yasser Fathi Hussein, Mona Riad

**Affiliations:** 1 Operative Dentistry Department, Faculty of Dentistry, Minia University, Minia, Egypt; 2 Department of Dental Materials, Faculty of Dentistry, Minia University, Minia, Egypt; 3 Conservative Dentistry Department, Faculty of Dentistry, Cairo University, Cairo, Egypt

**Keywords:** Dentistry, Bonded restorations

## Abstract

**Objective/aim:**

Evaluating the optical behavior and marginal discoloration of a Single-shade universal resin composite over 12 months. To achieve this, a split-mouth with a double-blinded randomized strategy was employed.

**Materials and methods:**

Twenty adult patients, each presenting with at least two caries lesions in their posterior teeth, were selected and randomly subdivided into two identical groups: Group I received Single-shade universal resin composite (Omnichroma), and Group II received multi-shade nanohybrid resin composite (Tetric®N-Ceram). Color measurements and marginal discoloration were assessed visually by three blinded operators at baseline followed after 1,3,6,9, and 12‑month periods utilizing the Modified United States Public Health Services (USPHS) criteria. Statistical analysis was adopted utilizing the Wilcoxon test with a 0.05 significance level.

**Results:**

The two groups revealed non-statistically significant differences up to 6 months regarding color match and color stability. After 9 and 12 months, the multi-shade group demonstrated a statistically significant higher prevalence of color match (Alpha) scores than the single-shade universal group. Regarding marginal discoloration, all restorations in the two groups had no discoloration (Alpha scores).

**Discussion/conclusion:**

Since the percentage of Alpha and Bravo scores was considered clinical success, both the single-shade universal and the multi-shade nanohybrid resin composites demonstrated satisfactory optical performance and marginal discoloration as posterior restorations after a 12-month follow-up period.

## Introduction

Due to the advancements in filler technology, resin composites (RC) have become a popular choice as aesthetic restorative materials for posterior and anterior teeth. Since tooth color is influenced by some aspects, including tooth type, location, and age, mimicking the resin composite color with the neighboring teeth was still challenging. Therefore, color-matching methods like utilizing composite materials of different shades that have been modified with dyes or pigments and employing multi-layered filling techniques and shade selection strategies [1].

The two main components of color changing in dental materials are the blending effect, which is primarily perceptual, subjective, and unmeasurable by any instrument, and a quantifiable, physically related translucency component. Dental materials that change color to match the surrounding hard dental tissues offer clinical advantages. This phenomenon can enhance the restoration’s esthetic appearance, facilitate shade matching, minimize the need for shade guide tabs, and eliminate the possibility of color mismatch [2]. Efforts have been made to develop dental materials that closely resemble the natural color of healthy teeth. One example of such a material is single-shade structurally colored universal resin composite, produced to achieve superior color matching natural tooth shades. This material is available in a single shade and aims to match the surrounding shade once it is placed and cured [3].

Nanohybrid resin composites are highly favored for their superior mechanical and physical properties and cosmetic appearance. This preference stems from their unique composition, combining nanoparticles with submicron size, resulting in improved filler distribution within the matrix [4]. These composites supplied with various shades and translucencies are typically categorized as enamel or translucent and dentin, opaque or body, intending to reproduce the dentin’s and enamel’s optical characteristics. It demands much clinical chairside time and is technique-sensitive to accurately mimic the exact color of the adjacent tooth. This need for simplifying shade selection has led to further investigations, leading to the establishment of the universal shades of composite [5].

Polymerization shrinkage in composite resins can lead to potential microleakage between the resin and tooth structure, which is a disadvantage. This microleakage allows the passage of irritants that can cause pulp inflammation, secondary cavities, post-operative sensitivity, and marginal discoloration. The procedure utilized to prepare the cavity, the restoration method, the polishing and finishing procedure, and outside elements like the patient’s diet and beverage can all impact the composite resin restorations’ marginal discoloration [6].

Laboratory investigations are used for the initial evaluation of any restorative material, but clinical studies are more crucial for evaluating its effectiveness. Therefore, several factors, including mastication stresses, temperature changes, humidity changes, and salivary enzymes, may impact the overall efficacy of restorative material [7]. Dental restorations are evaluated using a variety of clinical criteria. The most widely applied set of criteria is the Ryge criteria, also called “the United States Public Health Service (USPHS) criteria” [8].

However, this research intended to explore the optical behavior and marginal discoloration of single-shade structurally colored universal RC in posterior teeth over 12 months. The study’s null hypothesis was that the optical behavior and the marginal discoloration of new single-shade structurally colored universal RC are similar to multi-shade nanohybrid RC based on the modified USPHS criteria.

## Materials and methods

### Sample size calculation

The sample size calculation was based on the clinical success rate of composite restorations from prior research, which reported a rate of 93% at 12 months [9]. Previous research that examined posterior tooth restorations with a 0.05 significance level, power of 80%, and an equivalency limit of 20% used a sample size of 18 restorations. Considering the possibility of dropouts, we aimed to achieve a sample size of 20 restorations in each group, totaling 40 restorations due to the split-mouth strategy.

### Ethical approval and protocol registration

The study protocol was registered on www.clinicaltrials.gov (ID: NCT05500547). All procedures carried out on human participants followed the ethical standards of the Research Ethics Committee of the Faculty of Dentistry, with a serial number of 420 (Ref. no. 27 / 06 / 2020). The participants signed a written informed consent after receiving a detailed explanation of the process and the study’s objectives.

### Trial design and setting

The current study was a longitudinal, prospective randomized clinical trial (RCT) conducted as a split-mouth, with the clinical examiner and volunteers being double-blinded. It was done at the Operative Dentistry Department clinic, Faculty of Dentistry, between June 2020 and August 2021. The study’s reporting adhered to the Consolidated Standards of Reporting Trials (CONSORT) [10].

### Eligibility criteria

Participants in this study had to be healthy men and women patients of 20–45 years old. They were required to have at least two molars or premolars with occlusal carious lesions, where the size of the cavities was less than one-third of the intercuspal distance. These teeth were to be restored using two different types of composite resin. Inclusion in the study required the absence of any signs of fistula, pulp exposure, periodontal tissue swelling, or abnormal tooth mobility, as determined by radiographs or clinical examination. Participants were also expected to maintain acceptable oral hygiene and have healthy gingival tissues with no recession or alveolar bone loss. They should consent to return for evaluations.

Patients were disqualified from participating in the study if they were heavy smokers or had harmful parafunctional behaviors such as bruxism or traumatic occlusion. Additionally, individuals with natural opposing teeth that had existing restorations were also excluded. In addition, taking antibiotic within the last 6 months or the use of analgesics that could affect normal pain sensitivity led to ineligibility. Also, pregnant women and other patients who were medically compromised and had been given therapeutic radiation to the neck and head region were excluded.

Each participant signed a consent form before participating in the present study to ensure his knowledge about participating in this study and that he was informed about its nature.

### Randomization and allocation concealment

A split‑mouth Design was performed using (www.randomization.com), a computer‑generated block randomization technique, to randomly allocate the teeth into the two treatment groups. Every patient was given two class I posterior restorations, one on each side of the mouth. For each pair, one restoration was done with Omnichroma universal composite (test group) and the other with standard Tetric-N-Ceram composite (control group). The cavities were selected to match in dimensions and position within the tooth pair. The outcome assessor and data analyst were blinded to the allocation, whereas the operator could not be blinded since the control group requires shade selection [11].

### Cavity preparation

In a total of 20 individuals, 40 cavities were prepared. Periapical radiographs, vitality tests, and pre-operative pictures were taken and assessed before restorative treatment to complete the evaluation for inclusion in the research. To ensure minimal discomfort, anesthesia was administered prior to procedures, using 1.8 mL of 2% lidocaine hydrochloride with 1:2500 phenylephrine (SS White 100, SS White, Petropolis, Brazil). Shade selection was made for the control group, and rubber dam (Ivoclar Vivadent’s OptraDam® Plus) isolation was then applied to the treated teeth in both groups to avoid moisture contamination. The preparation of conservative cavities involved using a #330 high-speed carbide bur (FG, Dentsply Midwest®, Germany) with water coolant. The residual caries were then removed utilizing tungsten carbide burs (Komet, Brasseler GmbH Co. KG) at a reduced speed, along with sharp excavators (mailfaire 57/58, Switzerland). The preparation of the cavity was restricted to the elimination of caries, and the precise size and form of the cavity were achieved only upon the complete removal of carious tissue. Teeth with very deep cavities were excluded. Subsequently, the cavity walls were finished using yellow-coded stones (Komet, Germany).

### Application of restorative materials

Table 1 provides a description of the materials employed in the restorative procedure. A 36% phosphoric acid gel was applied to etch the enamel for 30 sec selectively, then rinsed and gently air-dried. The adhesive was applied to the prepared surface using a disposable brush then scrubbed for 20 sec, followed by gently air-drying using oil-free airflow for 3–5 sec and then light-cured using the light curing unit for 20 sec (Bluephase Style, Ivoclar-Vivadent, 1100 mW/cm2). Both resin composite restoratives were packed into prepared cavities using a composite applicator in 2 mm increments in the form of oblique layers, and each increment was cured for 20 sec. The restorations were finished and contoured simultaneously using low-speed fine-grit diamond finishing stones (Komet, Brasseler GmbH Co. KG) and copious water. Furthermore, the occlusal morphology was established using articulating paper (Bausch, Nashua, NH, USA). The polishing procedures were performed immediately using EVE DIACOMP Plus OccluFlex-impregnated rubber cups and impregnated brushes (Optishine, Kerr Switzerland) with water coolant and Soflex discs (3 M ESPE) in a recommended order (coarse, medium, fine, and superfine) with water coolant to obtain a smooth surface. All teeth were handled via one operator from the research team.

**Table 1:** Materials’ composition, manufacturers, and LOT numbers.

Material	Composition	Manufacturer	Lot #
Omnichroma	Matrix: TEGDMA^(1)^, UDMA^(2)^, Dibutyl hydroxyl toluene and UV^(3)^ absorber, Mequinol. Filler system: SiO_2_, ZrO_2_ (68 vol.-%; 79 wt%; 0.2–0.4 μm)	Tokuyama Dental, Tokio, Japan www.tokuyama-dental.com	002E60
Adhesive system: Palfique bond	Phosphoric acid monomer, Bis-GMA, TEGDMA^(6)^, HEMA^(7)^, camphorquinone, alcohol, and purified water.		176EY0
Tetric® N-Ceram Nano-hybrid incremental composite	Matrix: BisGMA^(4)^ and DMA^(5)^ (19-20 wt. %). Filler system: ytterbium trifluoride, barium glass, mixed oxide, and Copolymers (80-81 wt. %). Additives, stabilizers, catalysts, and pigments are included in dental materials as supplementary components, and their proportion is usually less than 1 wt. %. The dental material comprises approximately 55–57 vol. % of inorganic fillers, with a particle size ranging from 40 nm to 3000 nm.	IvoclarVivadent, Schaan, Liechtenstein www.ivoclar.com	Z0158P
AdheSE adhesive: Tetric N-bond Universal	Bis-GMA 25–50%, ethanol 10- < 25%, phosphonic acid acrylate 10- < 25%, urethane dimethacrylate 3- < 10%, diphenyl (2,4,6- trimethyl benzoyl) phosphine oxide 1- < 2.5%		Z00B7M

### Clinical procedures

#### Clinical assessment

Once the teeth were restored, the rubber dam was released, and the tooth was given 60 min until rehydration occurred [12]. Three calibrated blinded assessors clinically assessed the composite restoration’s color visually using both dental and natural light sources. They were pre-calibrated at 90% reliability on 10 patients who were not included in the study. The agreement between the examiners was observed to be substantial to almost perfect. Color measurements were made following USPHS criteria for a color match and color stability as follows: Alpha: The restoration closely resembles the translucency and color of neighboring dental tissues. Bravo: The color and translucency of the restoration show a minor deviation from adjacent dental tissues, but it falls among the typical range of tooth shades. Charlie: The restoration exhibits a significant discrepancy in shade and translucency when compared to the adjacent teeth structure, and the difference exceeds the typical range of tooth shade and translucency. Marginal discoloration assessed using a magnifying loupe with magnification power 3.5 as follow: Alpha: No marginal discoloration observed, Bravo: Marginal discoloration was present but limited in scope and did not extend significantly, Charlie: Noticeable marginal discoloration, penetrating towards the pulp chamber, Restorations were appraised at baseline, 1,3,6,9 and 12 months. The Alpha + Bravo score percentage was considered a clinical success.

## Results

### Color match

See Fig. 1.

**Fig. 1:**
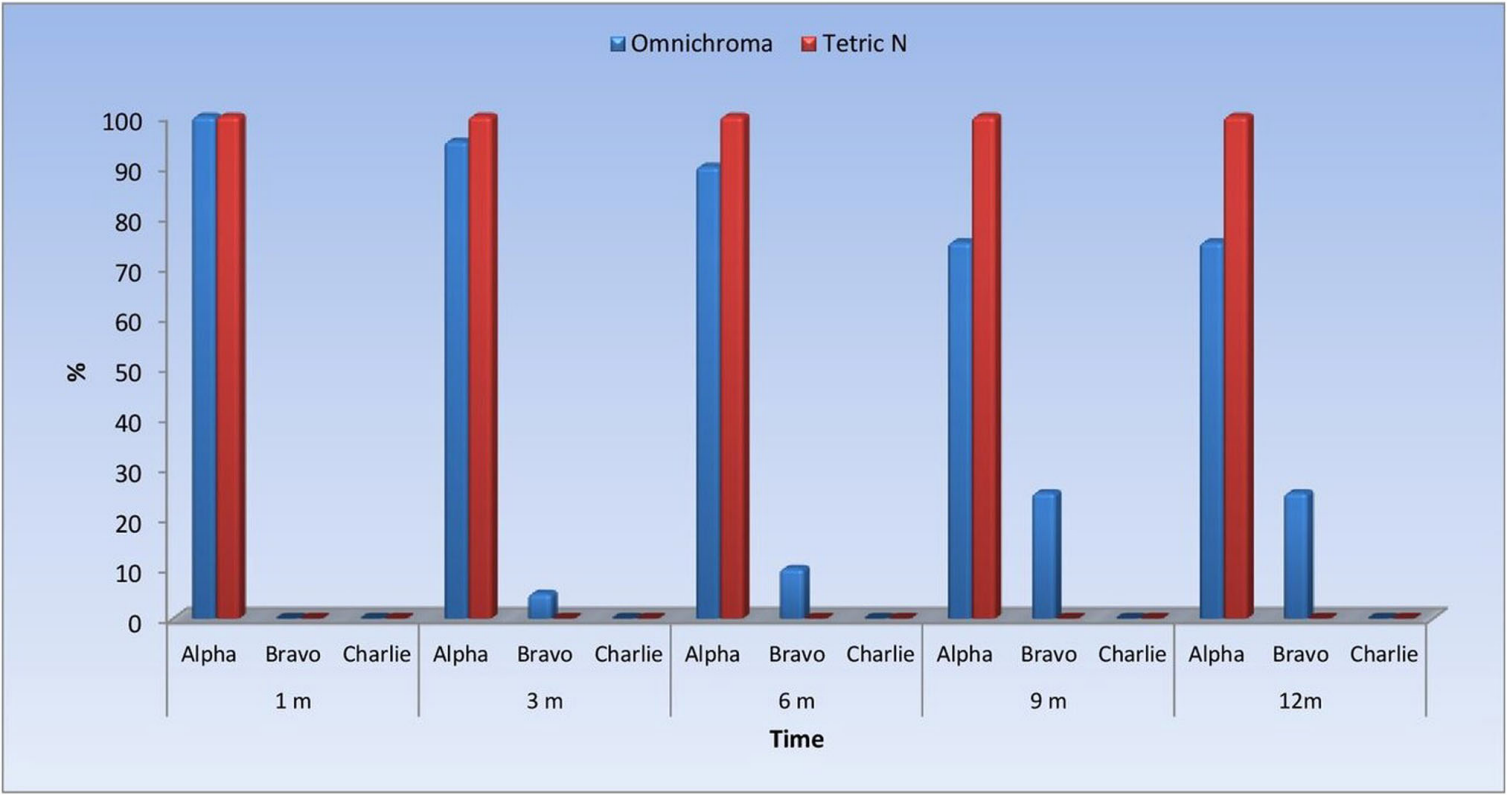
Bar chart representing color match scores in the two groups. Blue bars represent Omnichroma and red bars represent Tetric®N-Ceram.

### Comparison between groups

At the beginning (baseline), all restorations in the two groups revealed (Alpha) scores. After one, three, and six months, no statistically significant difference between both groups has been noticed (*P* > 0.05). After nine and twelve months, the Tetric N group displayed a statistically significantly higher prevalence of (Alpha) scores and a lower prevalence of (Bravo) scores than the Omnichroma group (*P* < 0.05) (Table 2).

**Table 2:** The findings of the Wilcoxon signed-rank test and descriptive statistics for comparison between color match scores in the two groups.

Time	Parameter	Material	Scores			*P*
			Alpha, *n* (%)	Bravo, *n* (%)	Charlie, *n* (%)	
Baseline	Color match	Omnichroma	20 (100)	0 (0)	0 (0)	1
		Tetric N	20 (100)	0 (0)	0 (0)	
1 month	Color stability	Omnichroma	20 (100)	0 (0)	0 (0)	1
		Tetric N	20 (100)	0 (0)	0 (0)	
3 months	Color stability	Omnichroma	19 (95)	1 (5)	0 (0)	0.317
		Tetric N	20 (100)	0 (0)	0 (0)	
6 months	Color stability	Omnichroma	18 (90)	2 (10)	0 (0)	0.157
		Tetric N	20 (100)	0 (0)	0 (0)	
9 months	Color stability	Omnichroma	15 (75)	5 (25)	0 (0)	0.025^a^
		Tetric N	20 (100)	0 (0)	0 (0)	
12 months	Color stability	Omnichroma	15 (75)	5 (25)	0 (0)	0.025^a^
		Tetric N	20 (100)	0 (0)	0 (0)	

^a^Significant at *P* ≥ 0.05.

### Changes within each group

The Omnichroma group exhibited a statistically significant change in color match scores over time (*P* < 0.05). The prevalence of (Bravo) scores increased after one, three, six, and nine months. However, no change was observed from nine to twelve months. In contrast, the Tetric N group revealed no statistically significant change in color match scores (Table 3).

**Table 3:** Descriptive statistics and findings of Friedman’s test for comparison between color match scores to the natural teeth at various follow-up periods within each group.

Material	Parameter	Time (months)	Scores			*P*
			Alpha, *n* (%)	Bravo, *n* (%)	Charlie, *n* (%)	
Omnichroma	Color stability	1	20 (100)	0 (0)	0 (0)	0.004^a^
		3	19 (95)	1 (5)	0 (0)	
		6	18 (90)	2 (10)	0 (0)	
		9	15 (75)	5 (25)	0 (0)	
		12	15 (75)	5 (25)	0 (0)	
Tetric N	Color stability	1	20 (100)	0 (0)	0 (0)	Not computed^b^
		3	20 (100)	0 (0)	0 (0)	
		6	20 (100)	0 (0)	0 (0)	
		9	20 (100)	0 (0)	0 (0)	
		12	20 (100)	0 (0)	0 (0)	

^a^Significant at *P* ≥ 0.05.

^b^Not computed because the variable is constant.

### Marginal discoloration

**Fig. 2:**
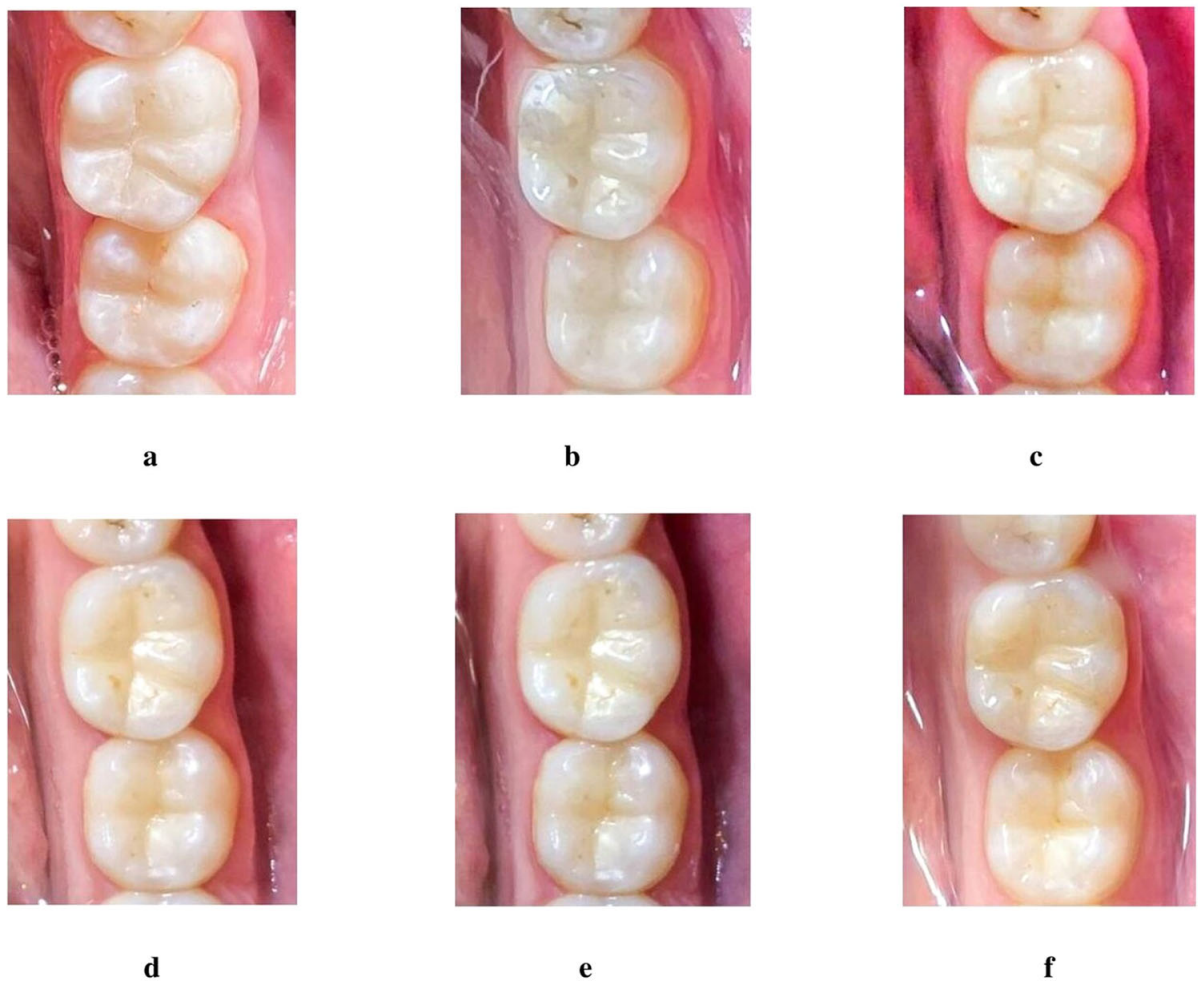
Clinical photographs of teeth 46 and 47 treated with Omnichroma and Tetric-N-Ceram, respectively. **a** At baseline photograph; **b** 1-month follow-up photograph; **c** 3-month follow-up photograph; **d** 6-month follow-up photograph; **e** 9-month follow-up photograph; **f** 12-month follow-up photograph.

All restorations in the two groups had (Alpha) scores, so no statistical comparison was performed (see Fig. 2).

## Discussion

Studying the optical behavior and marginal discoloration of single-shade structurally colored universal RC in posterior teeth offers essential insights for clinicians. These findings enable clinicians to support the use of dental materials that mimic and match natural tooth shades. This simplifies the shade selection and reproduction, ensuring patient satisfaction and acceptance of the dental esthetic treatment. Few studies have investigated the color adjustment potential of Omnichroma, and currently, there is limited evidence of its in vivo performance. Therefore, the objective of the current clinical trial was to evaluate and compare the clinical effectiveness of Omnichroma with Tetric‑N‑Ceram in permanent posterior teeth. Omnichroma® was selected due to its smart chromatic technology, which allows it to control its optical properties. This is achieved through a filler particle arrangement that matches the visible light wavelengths, thereby reducing color distortion and minimizing shade shift over time. This is possible due to decreased photochemical degradation [11, 12]. A nanohybrid composite Tetric N-Ceram was selected as the nano-fillers contain nanomodifiers, such as nanomers and nanoclusters, that modulate its mechanical and physical characteristics [13, 14].

Our results revealed that the color-matching capability of Omnichroma, a single-shade composite, was comparable to that of Tetric‑N‑Ceram, a multi‑shade composite. This may be due to the presence of uniformly sized and shaped supra-nano-filled particles, significantly contributing to a harmonious shade match. Omnichroma distinguishes itself by not containing any pigments; instead, it relies on structural color technology to govern the resin composite’s optical characteristics. This perspective has facilitated the development of a resin composite that reacts to light waves of a particular frequency via flawlessly reflecting a discrete wavelength within the tooth color spectrum [12]. The composite filler must contain only uniform, spherical particles with precise dimensions to exhibit structural coloration. Tokuyama’s study determined that 260 nm spherical filler produces the a and b color parameters required to mimic natural tooth shades. Variances in filler dimension and morphology can disrupt or hinder the structural color effect and, thus, the composite’s shade-matching capacity. Consequently, Omnichroma solely utilizes 260 nm spherical filler (OMNICHROMA Filler) material [15].

This study conducted to small sample size because it was difficult to convince patients to participate in this project as the study was done through the Covid-19 period. While the class I restorations were selected to easily standardize the cavity dimensions and C-factors.

In the current study, visual color match evaluation of any color change and stability of the color were ranked using the following scores based on the USPHS criteria, which ranked any color change on the following scale: Alpha: The restoration matches the color and translucency of neighboring dental tissues. Bravo: The color and translucency of the restoration show a minor deviation from adjacent dental tissues but still fall within the typical range of tooth shades. Charlie: The restoration exhibits a significant discrepancy in shade and translucency when compared to the adjacent teeth structure, and the difference exceeds the typical range of tooth shade and translucency, indicating a significant change.

No significant difference was found between both tested materials at 1,3, and 6 months recall periods. Comparatively, a significant difference was found in the color match and marginal staining at the 9- and 12-month recall periods, indicating the deterioration of the color of the Omnichroma restorations over time. While some of the single shade structurally colored universal RC restorations started to show a change in the Bravo score after the initial 9-month evaluation period, this was not regarded as a failure, and the change was clinically acceptable since the Alpha and Bravo score percentage was considered a clinical success [16].

These outcomes were verified by the study conducted by Ebaya and others, who indicated that Universal composites displayed acceptable surface smoothness and good color matching with various tooth colors [17]. Additionally, the RCs intelligent chromatic technology reflects the colors ranging from red to yellow, which are present in all teeth. This novel technology depends upon fillers composed of uniform supra-nano round and spherical particles made from zirconia and silica. These fillers are believed to produce structural color in the range of red to yellow when light penetrates the fillers. An unparalleled color matching is achieved by combining these colors with the surrounding tooth color. As a result, the composite blends with the surrounding tooth structure after curing. These findings align with Sensi et al., who demonstrated that Omnichroma showed minimal discoloration when subjected to artificial aging [18].

This result contradicts the results of de Abreu et al. and Iyer et al., who indicated that single-shade composites exhibit lesser color matching than multi-shade composites. This limitation can restrict their clinical usage in situations with high aesthetic requirements [19, 20]. The translucency parameter (TP), which can be enhanced to better match the resin composite with the nearby tooth structure in anterior restorations, is a significant concern despite the possibility that this resin composite will induce blending similar to various shades suggested by these findings. To address this issue, the manufacturer offers a “blocker” that can be utilized for class III restorations and compensate for the dark background of the oral cavity.

Contradictory results were demonstrated by Bajabaa et al., where Omnichroma showed the highest microleakage compared to Tetric‑N‑Ceram. This may be ascribed to the incorporation of TEGDMA within the resin matrix of Omnichroma, which has a low molecular weight compared to Bis‑GMA and UDMA in Tetric‑N‑Ceram, which considerably reduces polymerization shrinkage and microleakage. However, it is important to note that microleakage can lead to water sorption, stain penetration, and eventual discoloration [21].

Furthermore, Ebaya et al. [17] reported that the aging technique had a detrimental influence on the surface properties and color stability of universal composites. It is presumed that water absorption influences composites’ mechanical characteristics, leading to hydrolytic decomposition. Furthermore, water absorption may result in micro-fractures at the interface between the fillers and the resin matrix. High-temperature gradient variations near the surface can also induce superficial stress, affecting the surface’s roughness and ability to absorb stains.

Clinically, Omnichroma performed similarly to Tetric‑N‑Ceram with excellent color match and good color stability over 12 months, proving the null hypothesis. Hence, this novel single‑shade resin composite can be employed as an alternative to multi‑shade nanohybrid composite.

## Limitation

One of The study’s main limitations is that 12 months may not be enough time to detect significant alterations. So, a lengthy clinical assessment may provide a more accurate evaluation of the color stability of one-shade universal RCs.

## Conclusion

One shade universal composite resin exhibited comparable performance to a multi‑shade nanohybrid composite. The optical performance for color matching and color stability was reduced over time, however the change was deemed clinically acceptable.

## Data Availability

The datasets used and/or analysed during the current study are available from the corresponding author on reasonable request.
